# Glucosamine/chitosan blend surface-engineered rutin-loaded polymer/lipid hybrid nanoparticles for neuroprotection in induced schizophrenia model

**DOI:** 10.1038/s41598-026-52920-x

**Published:** 2026-06-17

**Authors:** Abeer Salama, Rania Elgohary, Alaa H. Salama, Heba Elmotasem

**Affiliations:** 1 Pharmacology Department, Medical Research and Clinical Studies Institute, National Research Centre (NRC), 33 El Buhouth St., Dokki, Cairo 12622 Egypt; 2 Narcotics, Ergogenics and Poisons Department, Medical Research and Clinical Studies Institute, National Research Centre (NRC), 33 El Buhouth St., Dokki, Cairo 12622 Egypt; 3 Department of Pharmaceutics and Industrial Pharmacy, Faculty of Pharmacy, Ahram Canadian University, 6th of October City, Cairo Egypt; 4 Department of Pharmaceutical Technology, Pharmaceutical and Drug Industries Research Institute, National Research Centre, Dokki, Cairo 12622 Egypt

**Keywords:** Rutin, Chitosan, Schizophrenia, Glucosamine, Polycaprolactone, Nanoparticles, Surface functionalized, Cuprizone, NRG1, Myelin, Biochemistry, Biological techniques, Biotechnology, Drug discovery, Nanoscience and technology, Neuroscience

## Abstract

The objective of this investigation is to enhance the bioavailability and therapeutic activity of a hydrophobic flavonoid rutin, with reported neuroprotective potential, by developing an innovative drug delivery system. Rutin-loaded polymer/lipid hybrid nanoparticles comprising polycaprolactone and Geleol™ or Captex® were developed. They were assessed for entrapment efficiency, size, and zeta potential. The selected formula (F4) comprised equal ratios of polycaprolactone and Geleol™, revealed nanoparticle size (226.9 ± 38 nm) and a high entrapment efficiency value (78.84 ± 6.1%). A glucosamine/chitosan hydrochloride blend was used as a functional coating of F4. The studied formulations improved the release profiles of rutin, which were 33.60 ± 3.1%, 64.76 ± 5.8%, and 59.12 ± 4.3% for rutin, F4, and CF4, respectively. The efficacy of free rutin and the selected formulations was assessed using cuprizone-induced schizophrenia in mice. Behavioral, biochemical, and histopathological investigations demonstrated the therapeutic potential of rutin, which enhanced brain myelin basic protein (MBP) and neuregulin 1 (NRG1) levels, thereby restoring myelination. Rutin also stabilized dopamine and glutamate pathways, alleviating cognitive and neurochemical imbalances characteristic of schizophrenia. The designed rutin-loaded formulation proved more effective than the free rutin, and the functionalized formulation outperformed the uncoated formula. Collectively, these results underscore the promise of this delivery system, while further assessments for pharmacokinetics, brain uptake, and clinical investigations remain essential to verify its translational potential.

## Introduction

Demyelinating diseases affecting the central nervous system (CNS) are neurological conditions. They are characterized by the progressive deterioration and loss of myelin sheaths and the cells that create them (oligodendrocytes) within the white matter pathways of the CNS. It is accompanied by widespread initial synaptic loss and progressive axonal degeneration^1^. The clinical manifestations of these disorders encompass ataxia, spasticity, impaired dexterity, paraparesis or hemiparesis, visual impairment, and cognitive difficulties resulting from disturbed electrical conduction along axons due to myelin degradation^2^.

Schizophrenia is a chronic central nervous system disorder that leads to psychotic episodes and substantial disability for people across the globe. Epidemiological data indicate that the global rate of new cases is about 1%^3^. Furthermore, the overall population affected by this illness grew significantly, affecting millions worldwide^4^. Schizophrenia symptoms (positive, negative, and cognitive) involve many different neurotransmitters, but the psychotic elements seem to be caused by a presynaptic dopamine excess (not a deficiency). Research has demonstrated that dopamine neurons release the co-transmitters glutamate and gamma-aminobutyric acid (GABA) in addition to dopamine in a synaptic signal mode^5^.

A cofactor of multiple “cuproenzymes,” copper (Cu) plays a crucial role in various cellular functions; serious dementia results from alteration of levels of copper in the nervous system^6^. Cuprizone (CPZ) increases the excessive generation of reactive oxygen species (ROS) in the neural tissue, which in turn drives neuroinflammatory reactions. It also causes neurotoxicity and demyelination in many brain regions^7,8^.

Myelin basic protein (MBP) and neuregulin 1 (NRG1) are key participants in the pathogenesis of schizophrenia, and the condition is exacerbated by their dysregulation. Anomalies in myelin deficiencies, synaptic connectivity, dopamine signaling, the emergence of symptoms resembling schizophrenia, especially negative and cognitive symptoms, and the disruption of connectivity between brain regions are all associated with changes in NRG1 expression and dysregulation of MBP^9^. There are currently no effective therapies for these illnesses, and their genesis is poorly understood.

The dysregulation of NRG1 and MBP highlights the complex neuro-developmental aspects of schizophrenia, suggesting new therapeutic targets. This study investigates the natural drug rutin as a myelination enhancer and a regulator of synaptic plasticity.

Rutin is a treasured flavonoid with potential therapeutic properties, found in various plants^10^. It offers various medicinal benefits for pharmaceutical use, including anti-inflammatory, antioxidant, neuroprotective, antidiabetic, cardiovascular, and anticancer activities^11^. Rutin helps prevent neurodegenerative disorders by reducing cellular damage and regulating brain inflammation and oxidative stress^12,13^.

Animal studies indicate that rutin may enhance memory and cognitive function in models of Alzheimer’s and Parkinson’s diseases. It protects neurons from apoptosis and excitotoxicity, which contribute to the pathogenesis of neurological disorders^13,14^.

Rutin’s therapeutic potential is limited by poor solubility and low permeability, resulting in reduced bioavailability^15^. Nanoencapsulation of the drug has been shown to improve bioavailability, but challenges remain in optimizing release kinetics, establishing effective targeting strategies, and developing scalable, reproducible manufacturing techniques^16,17^.

Numerous strategies have been developed to integrate multiple desired features into one nanoparticulate drug delivery system. Such integration is unattainable with individual components. One approach involves developing hybrid nanoparticles that combine the preferred characteristics of a multi-component system^18^. To apply this strategy effectively, each component must be carefully selected for its specific role in the formulated design. This approach is intended to improve the therapeutic activity of the delivered hybrid nanoparticles^19^.

One of the emerging techniques is to combine the benefits of both solid lipid and polymeric nanoparticles, forming polymer/lipid hybrid nanoparticles. Integrating the physicochemical properties of biodegradable polymer with those of lipids allows tailoring the characteristics of the resulting hybrid nanoparticles, including size, morphology, drug loading, and release profiles^20^.

Selecting an appropriate polymer for carrier formulation is essential and depends on factors such as biocompatibility, toxicity, desired particle size, surface charge, and the properties of the encapsulated drug^21^. Polycaprolactone (PCL) is widely used in drug delivery systems and has attracted significant attention due to its biocompatibility and biodegradability^22^. PCL can be used alone or in combination with other polymers. It is inexpensive, has a low melting point, is soluble in many solvents, and offers good controlled-release properties and optimal drug-loading capacity^23^. These properties make it a potential material for drug delivery to challenging areas such as the brain. Overcoming the blood–brain barrier (BBB) is a key challenge in employing PCL polymers for effective drug delivery to brain cells. Passing such a bottleneck obstacle is essential for the effective management of severe psychiatric disorders^23,24^.

Surface modification and functionalization of nanocarriers is an intriguing progression for improving brain delivery efficiency^25^. Studies indicate that the surface-coating nanosystems are essential for tailoring their performance. The benefits of surface coating can be tailored to the nanoparticle and the coating materials. The selection of appropriate coating material can enhance selectivity, reduce random distribution, and minimize unwanted exposure, resulting in fewer side effects. Drug delivery potential is further enhanced through decorating the surface with targeting moieties. This enables the recognition and binding of nanocarriers to the intended cells, as well as the selective delivery of their cargo to the intended site.

In the current study, a blend of chitosan and glucosamine was selected to modify the surface of the developed polymer/lipid hybrid nanoparticles because of their reported distinct properties. Given the diverse properties of chitosan and its promising applicability as a biomaterial coating for nanoparticle coating in biomedical contexts. Multiple studies have shown that chitosan-coated nanocarriers enhance mucoadhesiveness, improve tissue penetration and cellular uptake, increase bioavailability, and enhance drug efficacy. Chitosan shows considerable promise as a nanocarrier for brain drug delivery. Several investigations have verified the neuroprotective efficacy of chitosan nanoparticles, showing superior results in animal models of neuroinflammation and neurodegeneration. Moreover, the surface modifications of these nanoparticles enhance the binding of certain ligands or molecules, hence increasing the precision of drug delivery to neuronal cells^26^. Kruczkowska et al. reported that chitosan-coated micelles enhance stability and bioavailability, can assist brain delivery effectively, and exert a neuroprotective role^27^.

Studies indicate the use of chitosan-coated nanocarriers to enhance drug delivery across the BBB. These nanocarriers improve the bioavailability and effectiveness of medicinal agents, making them a potential option for several CNS disorders. Research demonstrates that the chitosan coating substantially facilitates the transport of decorated nanoparticles to the brain.

The coating of nanoparticles with chitosan allowed for the reformulation of various nanocarriers that do not show substantial transportation to the brain^28^. Cortés et al. reported that the application of the chitosan coating technique has provided many prospects for the formation of nanoparticles with promoted brain passage and delivery^29^.

Likewise, the addition of glucosamine can complement further advantages to the intended functionalizing coating. Glucosamine is recognized for its ability to traverse the BBB. Zheng et al. reported that glucosamine may penetrate the blood–brain barrier and reach the cortex, striatum, and hippocampal regions. Most importantly, glucose transporters have been identified in neurons and demonstrate the highest affinity for glucosamine^30^. Its protective effects against protein aggregation-related diseases and its potential for treating neurodegenerative diseases are of interest^31^. It can alleviate symptoms of amyloid-related neurodegenerative diseases by balancing their metabolic pathways^32,33^. Recent findings suggest that glucosamine exhibits neuroprotective and anti-neuroinflammatory properties. The association between glucosamine use and dementia may be elucidated through several distinct mechanisms. It has the potential to ameliorate the hallmarks of certain amyloid-based neurodegenerative disorders^32^.

Dendrimers coupled with d-glucosamine improved BBB permeability and efficiently targeted brain malignancies. Glucosamine-modified carriers exhibited more endocytosis compared to their nonglucosylated functionalized counterparts, suggesting that glucosamine serves as a viable mediator for targeting glial tumors and enhancing drug delivery systems^34^. Also, micelles functionalized with glucosamine were investigated as a therapeutic drug delivery method aimed at targeting the brain. This method was intended to improve therapeutic efficacy by enabling more efficient distribution to the brain regions^35^.

Another study reported that the brain delivery of thymoquinone was improved by manufacturing poly (d-glucosamine) self-assembled nanovesicles for targeted delivery. This was demonstrated using fluorescence imaging, showing improved uptake in brain tissues^36^. Such nanocarrier functionalizing techniques have high prospects for brain delivery^37^.

The objective of this work is to develop an innovative functionalized rutin lipid hybrid nanoscale formulation for the effective treatment of schizophrenia. Considering the various merits of the above-mentioned surface-functionalization materials, the mucoadhesive and absorption-enhancing chitosan, along with the reported assisted brain delivery potential and neuroprotective attributes of glucosamine. To our knowledge, this is the first study investigating the combination of glucosamine and chitosan as a coating for lipid/polymer hybrid nanoparticles to improve the therapeutic effectiveness of rutin in schizophrenia.

## Materials and methods

### Materials

Rutin was obtained from Kahira Pharmaceutical Company, Egypt. Chitosan hydrochloride, Mwt ~ 50,000 Da, was obtained from Zhejiang Chemicals Corporation, China. Glucosamine was a kind gift from Mepaco, Egypt. Polycaprolactone (PCL, Mwt ~ 14,000 Da), polyvinyl alcohol (PVA), and cellulose dialysis membrane (molecular weight cutoff 14,000 g/mol) were all obtained from Sigma-Aldrich (St. Louis, USA). Geleol™ was gifted by Gattefosse Co., France. Tween® 80 was obtained from El-Nasr Co., Egypt. All employed solvents were HPLC-grade. Myelin basic protein (MBP), interleukin-6 (IL-6), dopamine, glutamate, *N*-methyl-d-aspartate (NMDA), and Neuregulin-1 (NRG1) ELISA kits were purchased from Sunlong Co., China.

### Methods

#### Preparation of rutin-loaded-hybrid NPs

Hybrid nanoparticles were prepared adopting the emulsion solvent evaporation technique. PCL, lipid (either Geleol™ or Captex®), and rutin (20 mg) were dissolved in a precise volume of the organic solution mixture (10 mL) composed of dichloromethane and methanol (1:1 v/v). The organic system was subsequently instilled by dropping into an aqueous phase (10 mL) containing PVA (3% w/v) using a syringe fitted with a 19-gauge needle for a duration of 10 min under homogenization (Heidolph Instruments, Germany) at 20,000 rpm. The produced emulsion was stirred for 2 h at 1000 rpm using a magnetic stirrer to evaporate the organic solvent from the nanoparticle system. Lipid-free nanoparticles were fabricated for comparison purposes, while enhanced formulations were fabricated by adding Tween®80 to the aqueous phase before the homogenization process. Table 1 lists the composition of both the enhanced and hybrid nanoparticles.

**Table 1:** The composition and physical characteristics (EE%, PS, ZP, and PDI) of the developed rutin formulations. Data denoted as mean ± S.D. (n = 3).

Formula	PCL (mg)	Geleol™ (mg)	Captex® (mg)	Cosurfactant	EE (%)	PS (nm)	PDI	ZP (mV)
F1	50	–	–	–	51.36 ± 7.5	727.2 ± 73	0.764	− 6.54
F2	50	–	–	Tween®	57.45 ± 10.5	608.8 ± 137	0.632	− 8.36
F3	25	25	–	–	60.25 ± 8.2	537.9 ± 91	0.728	− 7.15
F4	25	25		Tween®	78.84 ± 6.1	226.9 ± 38	0.495	− 18.2
F5	25	–	25	–	55.11 ± 12.6	562.1 ± 59	0.599	− 5.09
F6	25	–	25	Tween®	61.50 ± 5.3	317.4 ± 43	0.456	− 10.90

#### Characterization of rutin-loaded-hybrid NPs

##### Particle size analysis

Prepared formulations were adequately diluted with deionized water, and then they were assessed for their diameters and PDI using ZetaSizer Nano ZS (Malvern Instruments Ltd., UK).

##### Entrapment efficiency (EE)

The prepared hybrid nanoparticles samples were centrifuged at 8000 rpm for 45 min at 4 °C by a cooling centrifuge (Union 32R, Hanil Co., Korea). The collected supernatants after triple washes were spectrophotometrically scanned for the free unentrapped rutin at 266 nm. The percentage EE of rutin within the prepared hybrid NPs was computed using the following equation:

$$ EE \left( \% \right) = \frac{Amount\;of\;entraped\;rutin}{{Total\;amount\;of\;added\;rutin}} \times 100 $$

Based on the results obtained from particle size analysis and entrapment efficiency experiments, one formulation showing optimum characteristics was selected and subjected to coating and further studies.

#### Preparation of coated hybrid NPs

To enhance the mucoadhesive property and delivery potential of the formed hybrid nanoparticles, a chitosan hydrochloride and glucosamine blend was performed on the selected hybrid nanoparticle formulation. Hybrid nanoparticles were prepared as described in the previous section on the preparation of rutin-loaded hybrid NPs, where equal amounts of chitosan hydrochloride and glucosamine were added to the aqueous solution containing the surfactant mixture. The blended polymeric coating mixture concentration was 0.2% w/v.

#### Characterization of the coated hybrid NP formulation

The coated formulation was subjected to particle size analysis and entrapment efficiency evaluation as previously described, as well as the following assessments.

##### In-vitro rutin release

The experiment was carried out for the selected hybrid nanoparticles formulation and its coated form in comparison with the free rutin form. An in-vitro rutin release study was performed with the aid of a thermostatically controlled shaker (LK LAB, Korea) adjusted at 37 ± 0.5 °C and 50 rpm. A certain amount of rutin (1.5 mg) and its equivalent amount in the selected formulations were located in previously hydrated dialysis bags firmly tied at both ends. The release medium was 50 mL of 0.1 N HCl for 2 h, followed by PBS (pH 6.8) for an additional 6 h. The release medium contained 10% methanol to ensure suitable sink conditions. At predetermined time intervals, samples (2 mL) were withdrawn from the release medium and substituted with fresh release medium of equal volume. Withdrawn samples were spectrophotometrically assessed for the amount of released rutin at 266 nm. The same procedure was repeated for a rutin aqueous dispersion, with an equivalent drug amount. The AUC_0–8h_ of the investigated release profiles was calculated using the trapezoidal rule. Also, the similarity factor values (*f*_2_) between the in *vitro* release profiles of uncoated and coated formulations and the free drug were calculated using the following equation:

$$ f_{2} = 50 \cdot log\left\{ {\left[ {1 + \left( {1/n} \right)\mathop \sum \limits_{t = 1}^{n} \left( {R_{t} - T_{t} } \right)^{2} } \right]^{ - 0.5} \cdot 100} \right\} $$

*f*_2_ value greater than 50 indicates similarity and *vice versa*^38^.

##### Kinetics of drug release

The drug release mechanisms from the selected nanoformulations were elucidated by kinetic analysis of in vitro release data. The data obtained were fitted using zero-order, first-order, Higuchi, and Korsmeyer–Peppas equations. Linear regression analysis was performed on the release data in order to identify the appropriate release model, which was evaluated based on the regression coefficient (R^2^). The best-fit model was defined as the release model with the highest R^2^ value^39^.

##### Microscopic visualization

The microscopical features of the selected formulations in their uncoated and coated forms were visualized using a transmission electron microscope (TEM; Jeol, JEM-1230, Japan). A drop from the analyzed samples was deposited onto a copper grid and then stained with a 2% w/v solution of phosphotungstic acid. After drying, the sample was examined utilizing the microscope.

##### Fourier transform infrared spectroscopy (FTIR)

To evaluate potential chemical interactions, the FTIR spectra of the optimized rutin-loaded formulation and its individual components were assessed at the ambient temperature via Jasco FT/-4600 Spectrophotometer, ranging from 4000 to 400 cm^−1^.

#### Biological evaluation of anti-schizophrenic effect

##### Animals

Male Swiss mice weighing 20–35 g were obtained from the animal house of the National Research Centre, Cairo, Egypt. The mice were kept in plastic cages with filter covers under controlled conditions of 12 h of light and 12 h of darkness, with 50% humidity at 28 °C. The mice were fed a regular pellet meal and had unlimited access to drinking water. Before initiating the experimental procedures, animals were allowed a one-week acclimatization period. The study protocol was approved by the Medical Research Ethics Committee of the National Research Centre, Egypt (Reg. No. 13020254). All experimental procedures were performed in accordance with the Guidelines and regulations for the Care and Use of Laboratory Animals published by the National Institutes of Health (Publication No. 85–23, revised 1985) and complied with ARRIVE guidelines.

Animal welfare was prioritized throughout the entire study. Measures were implemented to minimize pain and distress whenever possible using appropriate humane practices. Animals were closely monitored regularly, initially once daily and subsequently twice daily as the experiment progressed. A qualified laboratory animal specialist conducted welfare assessments to ensure proper oversight.

#### Design of experiments

Six groups were formed by randomly assigning male Swiss mice, with each group consisting of 10 mice. For 6 weeks, Group 1 acted as the control group and was given normal chow. The positive control group was designated as Group 2, specifically the cuprizone (CPZ) group. Blank formula was administered to Group 3. Groups 4–6 received free rutin, F4 uncoated formula, and the coated formula CF4, respectively, in a dose of 50 mg/kg of rutin, orally^40^. For the 6 weeks, Groups 2–4 were administered a diet consisting of 100 g of chow mixed with 0.2 g of CPZ (0.2% concentration)^41^. In the final 2 weeks (weeks 5 and 6), treatments were delivered via oral gavage once daily.

#### Evaluation of the open-field activity and Y-maze behavioral tests

The open-field test is a well-established technique for assessing spontaneous locomotor activity (measured as the distance and speed of exploratory movement in a novel setting) and anxiety-like behaviors (measured as time spent in the open central area)^42^. The Y maze (three different arms: A, B, and C) was used in accordance with the study; each time the mouse entered an arm with all its limbs, the letter was noted. Alternations denote the repeated entry into three distinct arms within overlapping triplet sets (for example, ABCBACA = 3). The total number of arm entries is just the count of submitted arms (for instance, ABCBACA = 7). The following equation was used to compute the % alternation^43^:

$$ {\text{The percentage alternation}} = \frac{{{\text{Number of alternations}}}}{{({\text{Total arm entries}} - 2)}} \times 100 $$

##### Biochemical analysis of tissue

Using mild anesthesia with thiopental sodium (50 mg/kg, i.p), cervical dislocation was employed to euthanize six mice from each group^44^. Immediately after detachment, each mouse’s brain was cleaned using phosphate-buffered saline (PBS) to eliminate any excess blood. Components that were weighed were homogenized in PBS with the use of an MPW-120 homogenizer (Med Instruments, Poland) to create a 20% homogenate, which was stored overnight at − 20 °C. Centrifugation of the homogenates was performed at 5000×g for 5 min using a cooling centrifuge (Sigma and Laborzentrifugen, 2k15, Germany)^45^. The supernatant was promptly gathered and stored at − 80 °C. Using ELISA kits, levels of glutamate, dopamine, NRG1, NMDA, MBP, and IL-6 in the brain were measured^46^.

##### Examination of histopathology

Samples for autopsy were gathered from the brains of four mice in each study group and stored in 10% formalin saline for a duration of 24 h. Before dehydration with a series of alcohol dilutions, the samples were rinsed using tap water. Before being embedded in paraffin at 56 °C for 24 h in a hot air oven, the samples underwent cleaning with xylene. Using a rotary LEITZ microtome, tissue blocks made of paraffin bees’ wax were sectioned to a thickness of 4 µm. Glass slides were used to mount the acquired tissue slices, which were then deparaffinized and stained with hematoxylin and eosin for examination under a light microscope. An average brain section from each group was blindly examined using a semiquantitative scoring system; the inflammatory response and neuronal loss were graded as: 0, absence; 1, mild; 2, moderate; and 3, severe^47^.

#### Statistical evaluation of results

The values are presented as means ± SD. One-way analysis of variance (ANOVA) was utilized to make comparisons across groups, with Fisher’s LSD test applied for multiple comparisons afterward. Using GraphPad Prism software (Inc., USA), statistical tests were conducted. A significant level of *p* < 0.05 was considered.

## Results and discussion

### Rutin-loaded hybrid nanoparticles size

The main advantage of nanoparticles is their tiny size, which offers benefits concerning improved delivery. Thanks to their tiny size, nanoparticles can preferentially adhere to the inflammatory sites and demonstrate enhanced retention^48^. Careful selection of the surfactant mixture is a must^49^.

Table 1 depicts that the F1 formula, prepared employing polycaprolactone solely as matrix former, demonstrated the biggest particle size (727.2 ± 73 nm). The emulsification ability of PVA succeeded in producing particles in the nanometric range; however, the produced size is not satisfactory for our study aim. Whereas upon mixing PCL with the lipid component (Geleol™ or Captex®), in the hybrid formulations, a significant decrease in particle size was observed (537.9 ± 91 and 562.1 ± 59 nm, respectively). The size-reducing ability of the added lipids (Geleol™ or Captex®) could be explained by their amphiphilic nature and possession of low HLB-values^19,50^.

On the other hand, the addition of Tween® 80 as a co-surfactant exerted an additional effect in decreasing the surface tension of the formed particles. A significant decrease in particle size was observed with its usage. This also showed that both surfactants were compatible and did not interfere with each other's adsorption on the nanoparticle surface^51^. Tween® 80 was previously recorded as an effective co-surfactant in reducing the particle size of high-molecular-weight based polymeric structures^19^.

The HLB-value of Tween®80 (> 10) enhanced the emulsification power^52^. Moreover, the alignment of the hydrophobic monooleate segments of Tween® 80 with PVA molecules around the minute droplet surfaces significantly reduced the interfacial tension and, therefore, the particle size. Sharma et al. observed the same findings, indicating that the integration of Tween®80 as a co-surfactant was linked to a substantial reduction in particle size^53^. Moreover, the use of Tween®80 in brain nanoformulations offers additional benefits beyond its role as a cosurfactant. Tween® 80 was reported to have brain-targeting capabilities. Yadav et al. reported that Tween® surface-modified nanoparticles can effectively penetrate the BBB^54^.

### Rutin entrapment efficiency

The prepared formulations displayed high EE% values, above 50%, as demonstrated in Table 1. This indicates the ability of the system to entrap rutin within the formed NPs. This could also be related to the usage of the proper type and amount of the surfactant. Furthermore, it was found that the addition of Tween® significantly enhanced the entrapment of rutin within the fabricated nanoparticles. This reflects the compatibility of the surfactant/co-surfactant mixture and their high ability to entrap rutin. The addition of lipids to create hybrid structures significantly improved the drug EE%. Mixing Captex®200, the medium chain triglyceride, possessing amphiphilic characteristics within the nanoparticle matrix, positively enhanced the entrapment efficiency^55^. Additionally, the inclusion of Geleol™ significantly improved the entrapment of rutin compared to Captex®200. Geleol™ is a glyceride with an amphiphilic nature, having an HLB value of 3^50^. The results clearly showed that the EE % -values in hybrid formulations were significantly influenced (*p* < 0.05) by the HLB-value of the used lipid.

The higher HLB value of Captex®200 may facilitate rutin permeability from the formed nanoparticulate structures^49^.

Based on the obtained particle size and EE% efficiency results, the hybrid formulation (F4), which showed a particle size value of 226.9 ± 38 nm, ZP of − 18.2 mV, and the highest EE%-value (78.84 ± 6.1%), was selected for additional studies.

### Coated rutin-loaded hybrid nanoparticles

#### Particle size, PDI, zeta potential, and entrapment efficiency

The EE % of the CF4 formula was 80.2 ± 4.7. However, there was no significant difference (*p* > 0.05) for entrapment efficiencies of the selected formulation before and after coating. Particle size and PDI of coated rutin-loaded nanoparticles were 331.2 ± 32.5 nm and 0.71, respectively. The coated formulation PS was larger than the uncoated one, owing to the deposition of the coating layer around the rutin-loaded nanoparticles. The ZP value of the coated formulation was + 16.56 ± 1.9 mV. The inversion of the surface charge of the prepared nanoparticles, CF4, from a negative to a positive sign compared to the negative charge of the uncoated F4 formula, reflects the success of surface functionalization and deposition of the cationic (glucosamine/chitosan blend) on the nanoparticles' surfaces. Especially due to the presence of amine groups in both glucosamine and chitosan, which neutralize the negative charge of the uncoated particles and then further surmount it and render dominant positive charges after surface deposition on the nanoparticles. Moreover, the positively charged particles can enhance permeation through the negatively charged BBB^26^.

#### In-vitro rutin release

**Fig. 1:**
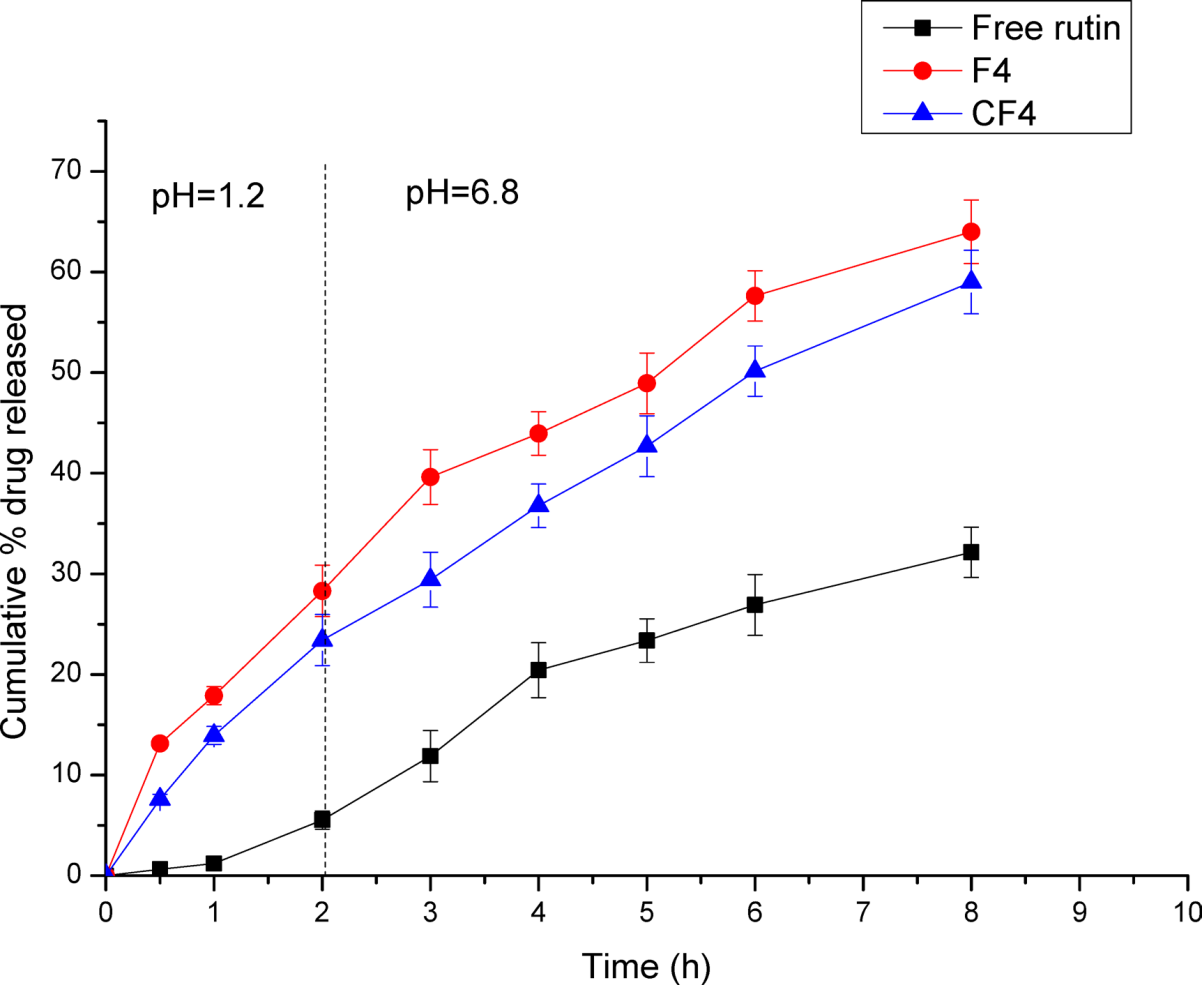
Effect of the investigated drug formulation on the cumulative % rutin released.

Observing the drug release profile is necessary to assess the efficiency of the designed nano-formulated drug. Figure 1 demonstrates the release profile of the selected hybrid formulation (F4), its coated form, in comparison to the aqueous dispersion of the drug. As manifested, only 33.61 ± 3.1% of free rutin was released through the cellulose membrane after 8 h. The selected formulations showed an enhanced rutin release compared to the free drug (*p* < 0.005), after 8 h with 64.78 ± 5.8% and 59.10 ± 4.3% of the drug released from the uncoated formula F4 and the coated CF4 formula, respectively.

Additionally, the AUC_0–8h_ values (Table 2) for the cumulative % release versus time for the free drug, F4, and CF4 were 144.9 ± 5.9, 326.1 ± 12.4, and 282.9 ± 7.9, respectively. The results revealed that both formulations, F4 and CF4, had significantly higher (*p* < 0.05) AUC_0–8h_ values compared to that of the free drug. This can be attributed to the nanoencapsulation of rutin, which increases its surface area and promotes its release over time. It was noted that CF4 showed a significantly smaller AUC_0–8h_ value compared to F4 (*p* < 0.05). This can be explained by the surface coating and the larger PS of CF4, which lead to a longer diffusion path that the drug must travel before reaching the release medium. This result indicates that CF4 exhibited a slower and more controlled release pattern.

**Table 2:** Release profile kinetic modeling for the investigated formulations.

Formula	Zero order	First order	Higuchi order	Peppas model	
	R^2^	R^2^	R^2^	R^2^	n
F4	0.940	0.984	0.991	0.994	0.596
CF4	0.951	0.984	0.976	0.987	0.740

The similarity factor (*f2*) value between the release % curve of the free drug and that of F4 was 23.24, and the f2 value between the free drug and the release % curve of CF4 was 29.98. These values were < 50, indicating dissimilarity of their release patterns. Moreover, the *f*2 value between F4 and CF4 was 49.46, indicating that their release patterns were dissimilar.

The release profile was subjected to kinetic modeling. The results (Table 2) revealed that the release mostly fit the Peppas model with the highest R^2^ value. The n-values for F4 and CF4 were 0.596 and 0.740, respectively. These results imply an anomalous type of release mechanism^56,57^.

Previous studies reported that three steps are involved in drug release from hybrid nanoparticles. At first, the weakly bound drug molecules are desorbed from the surface, and released to the medium^58^. Then, the drug is diffused from the polymeric matrix in a controlled pattern, along with the start of matrix degradation. At last, the mutual degradation/diffusion phase predominates^59^. This controlled drug release could be credited to the appropriate assortment of the utilized ingredients and their amounts in fabricating the hybrid nanoparticles matrix. Briefly, the penetration of the release medium in the formed hybrid polymeric matrix channels permits drug dissolution. This consequently forms a saturated drug diffusion layer, responsible for particles distension and gradual drug release into the bulk of the medium. This mechanism can be followed by slow matrix degradation.

This observed controlled rutin release from the designed hybrid delivery system may conserve adequate rutin concentrations in the blood for prolonged periods, and hence less frequent rutin administration as well as improved biological performance and patient compliance.

#### Visualization by TEM

**Fig. 2:**
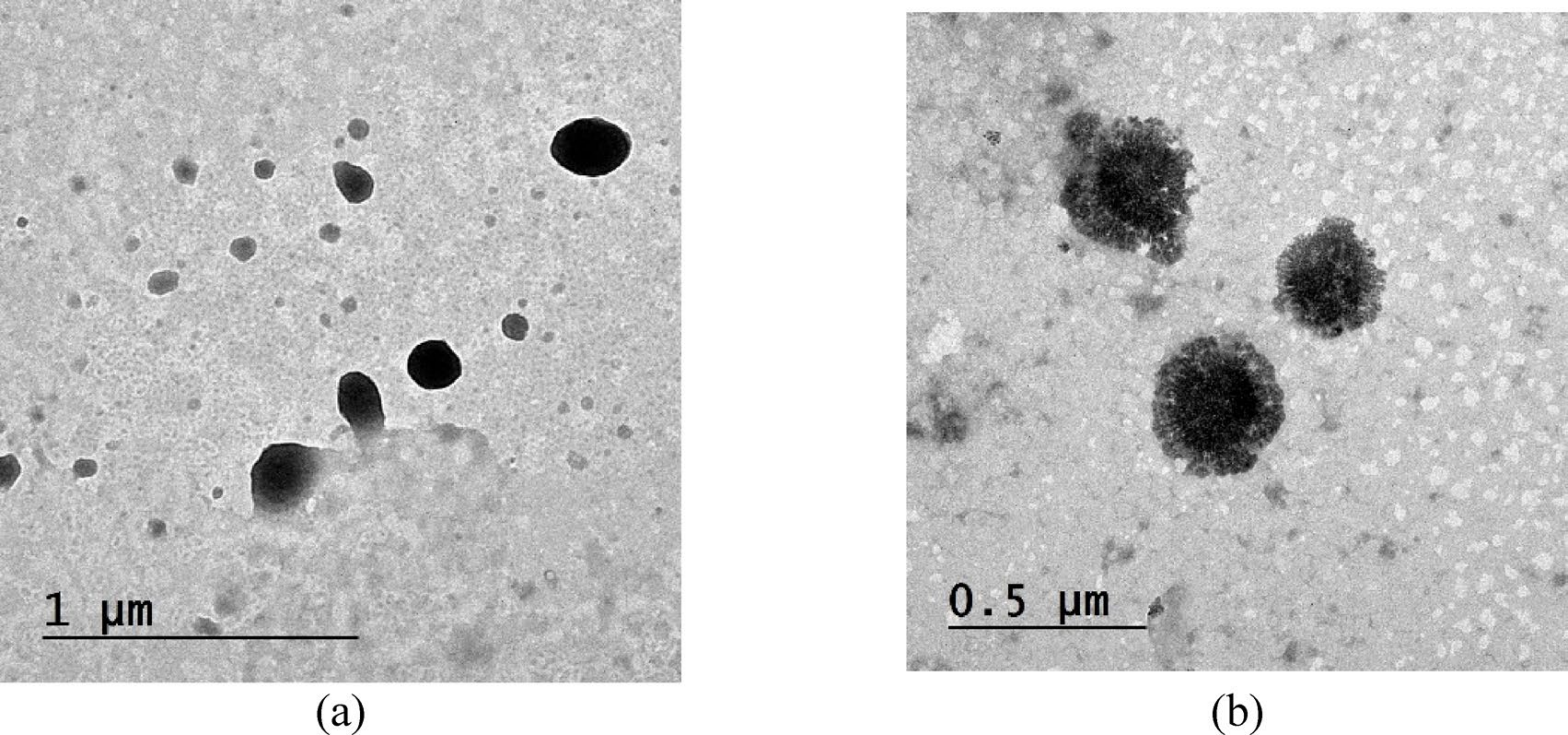
TEM micrographs for uncoated (F4) (**a**) and coated (cF4) (**b**) selected rutin-loaded nanoparticles.

The micrographs of the selected formulation in its coated and uncoated forms are demonstrated in Fig. 2. As shown in the figure, the examined formulation appeared as spherical structures.

The chitosan/glucosamine surface coating layer on the nanoparticles 4FC was apparent as a grey decorating sheath. This coating layer surrounding the particles of the selected formulation confirmed the success of the surface modification process. The particle size of the investigated formulations, as measured by TEM, corresponded well with the results obtained from ZetaSizer Nano ZS.

#### FTIR

**Fig. 3:**
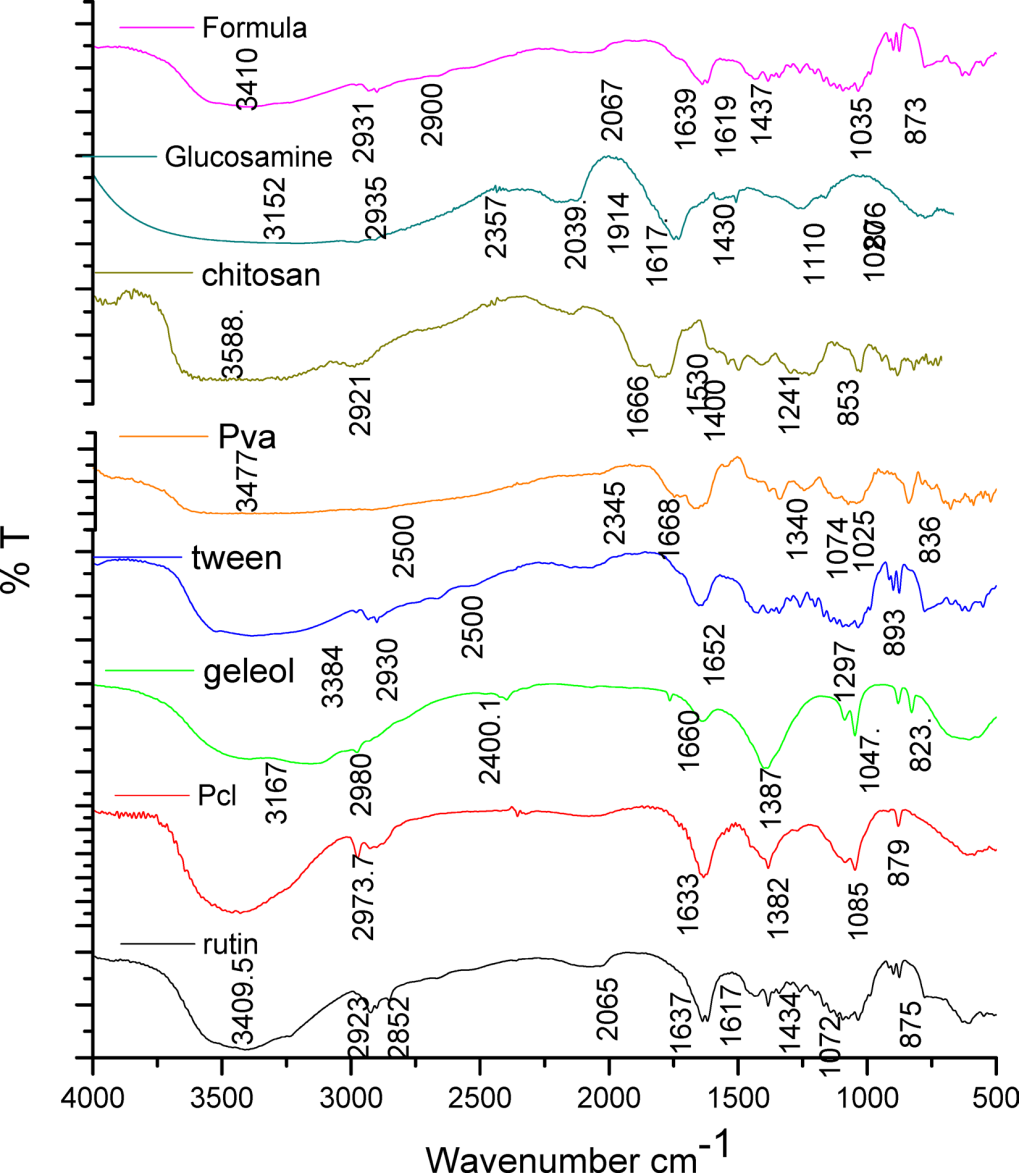
FTIR spectra for the selected formulation, along with formulation ingredients.

The FTIR spectra of the selected formulation and its constituents are used to assess potential interactions among the ingredients. The FTIR spectrum (Fig. 3) of rutin shows a band appearing at 3409.5 cm^−1^ due to OH stretching. The stretching vibrations absorption bands that appeared in the range 2923.56–2852.2 cm^−1^ are attributed to C–H groups. The bands at 1637.27 cm^−1^ and 1617.98 cm^−1^ result from C=O and C=C stretching vibrations. The bands of C–O–C and C–O are depicted at 1342.21 cm^−1^ and 1382.71 cm^−1^. The absorption bands at 1072.23 cm^−1^ and 1035.59 cm^−1^ are ascribed to the bending vibration of hydroxyl groups, and the band at 875 cm^−1^ is attributed to aromatic and aliphatic C–H vibrations.

The FTIR spectrum of polycaprolactone shows a distinctive absorbance peak at 2973.7 cm^−1^ arising from the symmetric and asymmetric stretching vibration of the C–H. The broad band at 3428 cm^−1^ is attributed to residual moisture. A strong peak for ester carbonyl stretching appeared at 1633 cm^−1^. The symmetric C–O–C stretching bands appeared at 1382 cm^−1^ and 1085.73 cm^−1^. The rocking vibration of the long –CH_2_– chains appeared at 879 cm^−1^.

Also, the spectrum of Geleol™ exhibits a broad band at 3167 cm^−1^, attributed to OH, while the band at 2980 cm^−1^ corresponds to CH stretching. The band at 1700 cm^−1^ is ascribed to the stretching of the ester carbonyl group. Bands in the 1637 cm^−1^ region correspond to in-plane C–H bending. Moreover, the band at 1047 cm^−1^ corresponds to the bending of C–O–C bonds. Alongside the bands corresponding to aliphatic CH vibrations in the fingerprint region, 820 cm^−1^.

The FTIR spectrum of PVA demonstrates broad and strong band at 3470 cm^−1^ region related O–H. The band at 2345 cm^−1^ reflects C–H and CH_2_ stretching vibrations. The bands at 1668.12 cm^−1^ and 1552.41 cm^−1^ are due to CH_2_ bending. Bands appearing at 1378.85 cm^−1^ and 1340.28 cm^−1^ are due to C–H twisting. The band that appeared at 1074.16 cm^−1^ indicated alcoholic C–O stretching group. The band at 838.98 cm^−1^ could be related to polymeric backbone C–C vibrations.

FTIR spectrum of Tween® shows a band at 3384 cm^−1^ assigned to sorbitan residual O–H groups stretching. The peaks at 2930 cm^−1^ and 2950 cm^−1^ are due to CH2 stretching. The bands at 1410 cm^−1^ are due to the bending of CH_2_ groups. The band at 1652 cm^−1^ arises from oleate C=C stretching vibration. The C–O ester linkage absorbance appeared as a band at 1100 cm^−1^.

Whereas the FTIR spectrum of chitosan revealed a strong, broad peak appeared in the 3500 cm^−1^ region due to the presence of NH_2_ and hydroxyl groups. The CH and CH2 groups' absorbance appeared in the 2900 cm^−1^ region. The peak appearing at 1666 cm^−1^ is related to the primary amine I C=O stretching. The bending vibration of N–H (amide II) appeared at the 1560 cm^−1^ region. The CH_2_ bending vibration band appeared at the 1430 cm^−1^ region. The bands appearing at 1100–1120 cm^−1^ are due to C–O and C–O–C of chitosan glycosidic polymeric backbone linking stretching vibration. The C–H deformation absorption band appeared at 850 cm^−1^.

The spectrum of glucosamine shows a wide, strong band that appears in the 3300–3000 cm^−1^ range, attributed to NH_2_ and OH groups. Bands appearing at 2900 cm^−1^ region pertain to C–H bonds, the peak appearing at 1617 cm^−1^ is due to the presence of secondary OH and amide I. The CH_2_ bending appears at the 1430 cm^−1^ region. The bands appearing at 1100 cm^−1^ are due to the stretching of the C–O group.

Finally, in the FTIR Spectrum of the optimized formula, the unique peaks of rutin and the other formula components were exhibited with minimal variation. This can affirm the non-appearance of chemical interactions between the formula excipients and the drug^60^.

### In vivo studies

#### The selected formulations impact on mice behavior, myelin, and basic protein (MBP) in the brain

Myelin deficiencies are associated with a variety of psychiatric diseases, including ADHD, psychosis, depression, and bipolar disorder^61^. The myelin sheath is essential because it maintains the structure and survival of axons, allowing nerve messages to be relayed rapidly and correctly^62^. Demyelination can occur in both congenital and acquired myelin diseases, such as multiple sclerosis, which leads to axon and neuron degeneration, as well as persistent neurological problems like dementia and schizophrenia. Schizophrenia is likely the most studied psychiatric condition in terms of white matter abnormalities, with the most reliable results. The condition is distinguished by altered perception of reality, most commonly manifested as disorganized mental processes and speed, visual, auditory, paranoid hallucinations, and/or social dysfunction^63^. CPZ significantly (*p* < 0.05) inhibited MBP brain content by 81% (3961.93 ± 258.85 vs. 20,720.70 ± 3922.93) as compared to normal, and induced recognition memory deficits^64^. In the current investigation, the treatment with free rutin, F4, and coated CF4 rutin treatments significantly (*p* < 0.05) caused a considerable rise in MBP brain content by 109%, 232% and 402% (8299.65 ± 697.80, 13,154.91 ± 2254.42, 19,887.37 ± 1862.36 vs. 3961.93 ± 258.85) respectively as compared to CPZ group, which is in line with a research which showed that rutin helps protect the myelin sheath in the sciatic nerves of rats that have been damaged by acrylamide. It achieves this by increasing the levels of MBP^65^.

**Fig. 4:**
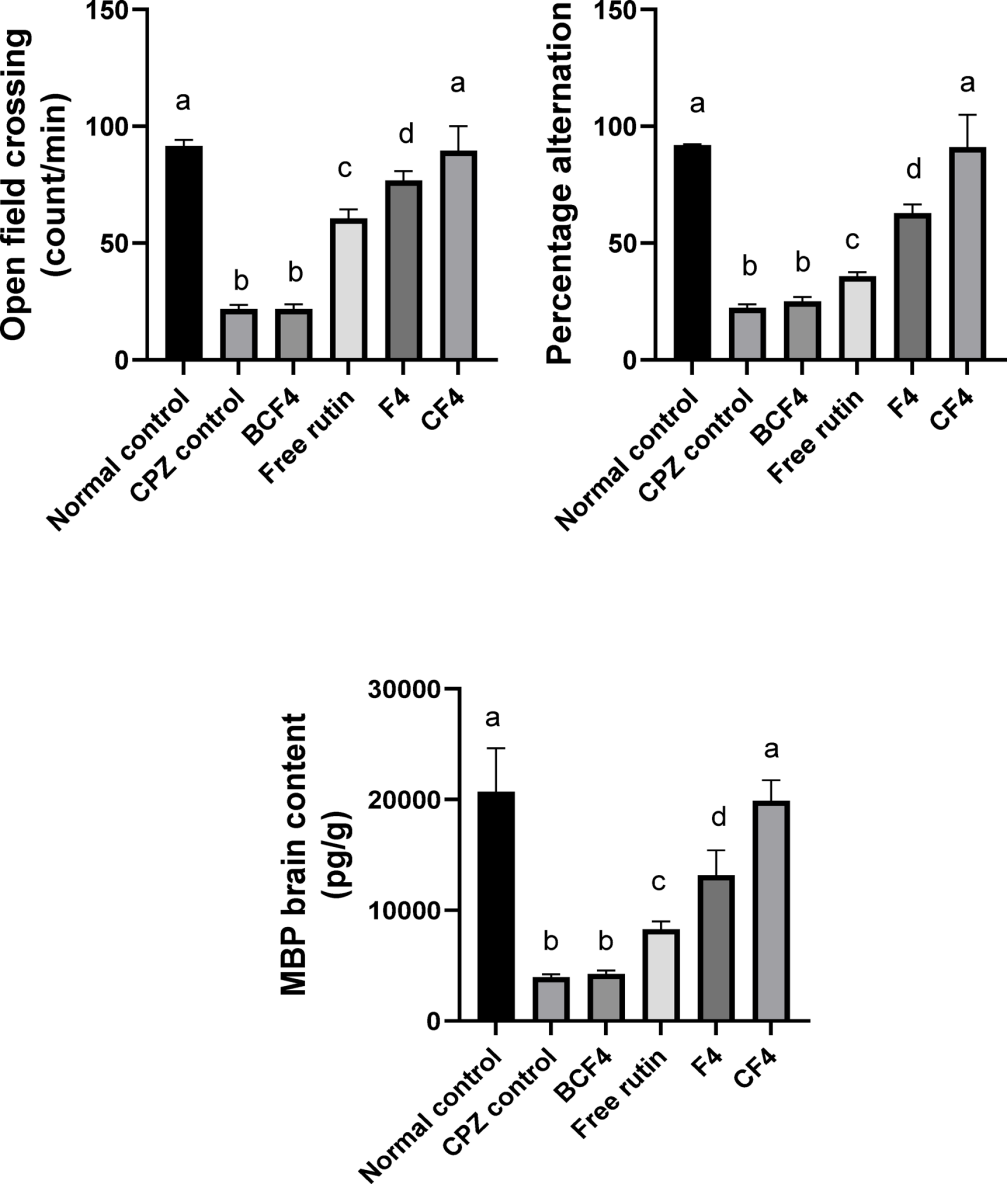
Effect of the free rutin, uncoated F4, and coated CF4 nanoformulations on mice behavior and myelin basic protein (MBP) in the brain. Identical letters indicate no significant difference between parameters, whereas differing letters signify a significant difference at *p* < 0.05.

Schizophrenia typically begins in the teens to twenties and is linked to cognitive deficits. Demyelination fosters chronic inflammation, leading to symptoms like anxiety and motor impairment. The CPZ model is widely used for its reliability, low cost, and ability to replicate some key features of schizophrenia. It is useful for studying demyelination and screening drugs that promote myelination. CPZ induces demyelination in the corpus callosum, causing motor coordination deficits^66,67^. Nevertheless, this model has its limitations because schizophrenia can arise from various factors, including genes, brain development, and chemical imbalances. In comparison, CPZ toxicity is caused by a chemical and only shows damage to the coating on nerves. Although some thinking and social problems show up with CPZ, this model does not show symptoms like hallucinations and delusions. Also, when CPZ is stopped, nerves can recover, which is not like the long-lasting, worsening course of schizophrenia^68^. It must be acknowledged that no single model can mimic all schizophrenia symptoms. However, the CPZ model induces demyelination in the corpus callosum, which results in motor coordination deficits^69^. Since schizophrenia often includes depression, agitation, and memory problems, both the Y-maze and OFT were used (Fig. 4). The Y-maze confirmed significant spatial memory deficits. CPZ significantly (*p* < 0.05) decreased % alteration by 76% when compared to normal (22.32 ± 1.44 vs. 92.99 ± 0.32). Rutin therapy improved poor motor coordination in CPZ mice (Fig. 4). The free rutin, the uncoated formula F4, and the coated formula CF4 therapy could significantly (*p* < 0.05) improve mice’s spatial working memory by enhancing spontaneous Y maze alternation by 59%, 181%, and 308% (35.83 ± 1.77, 62.78 ± 3.80, 91.01 ± 13.97 vs. 22.32 ± 1.44), respectively, as compared to the CPZ group, indicating better cognitive function. Furthermore, OFT data revealed that the mice exhibited anxiety symptoms^70^. However, CPZ increased anxiety by 76% (21.75 ± 1.79 vs. 91.50 ± 2.69) as compared to normal. The free rutin, the uncoated formula F4, and the coated formula CF4 therapy significantly (*p* < 0.05) reduced anxiety in mice by 178%, 253%, and 311% (60.50 ± 3.91, 76.75 ± 4.09, 89.50 ± 10.52 vs. 21.75 ± 1.79), respectively, as compared to the CPZ group, making them more active and balanced, implying that CPZ-induced demyelination may be ameliorated. These findings agreed with those of Nicola et al., who found that rutin therapy corrected behavioral abnormalities in a multiple sclerosis animal model^71^.

#### The selected formulations impact on IL-6, dopamine, glutamate, NMDA and NRG1

**Fig. 5:**
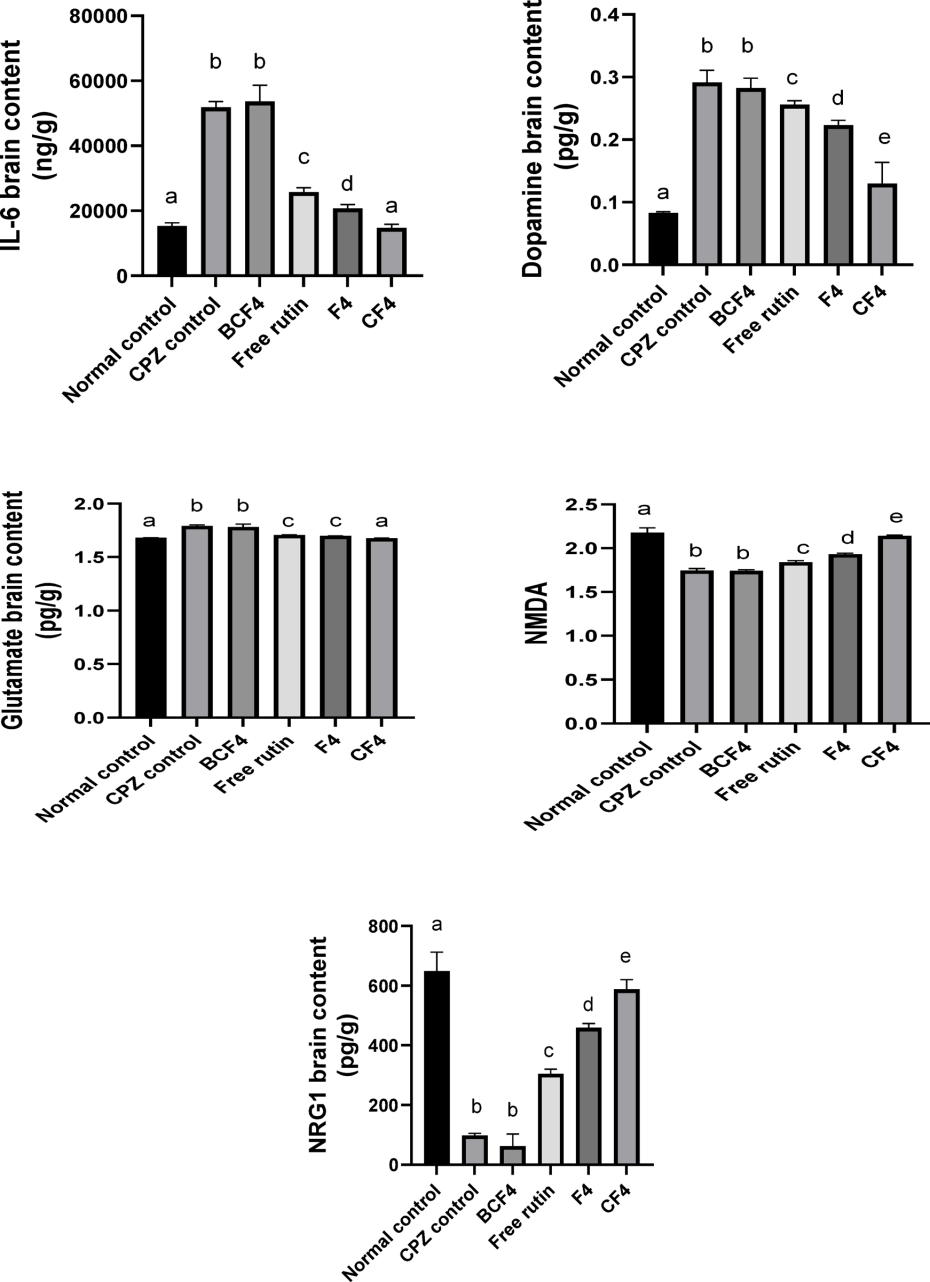
Effect of the free rutin, uncoated F4, and coated CF4 nanoformulations on the levels of IL-6, Dopamine, Glutamate, NMDA, and NRG1. Identical letters indicate no significant difference between parameters, whereas differing letters signify a significant difference at *p* < 0.05.

CPZ exposure is a newly established neuroinflammatory psychosis model that causes oligodendrocytic damage via mitochondrial dysfunction, as well as behavioral abnormalities such as hypersensitivity to psychostimulants and deficiencies in short-term mental tasks, which are most likely linked to elevated levels of proinflammatory IL-6 within the hippocampus^72^. Inflammatory cytokines generated by microglia and astrocytes that possess a cytotoxic role, as well as mitochondrial dysfunction, promote the production of IL-6, resulting in functional deficiencies that include accelerated demyelination, reduced myelin synthesis, disruption of synaptic plasticity, and are linked to cognitive deficits and behavioral abnormalities, features of schizophrenia^73^. CTZ significantly (*p* < 0.05) increased IL-6 by 238% (51,832.11 ± 1775.24 vs. 15,318.95 ± 951.38) as compared to normal. Interestingly, free rutin, F4, and coated CF4 rutin treatments alleviated neuroinflammation by considerably lowering IL-6 levels by 50%, 60%, and 72% (25,779.47 ± 1350.78, 20,792.63 ± 1104.95, 14,766.32 ± 1043.38 vs. 51,832.11 ± 1775.24), respectively, compared to the CTZ group, which is consistent with prior results on rutin in neurodegenerative disorders such as Alzheimer's and Parkinson's disease^74^. The current study is looking into how IL-6 restriction by rutin promotes remyelination, which could restore the cognitive and neurological impairments reported in CPZ-treated mice, possibly revealing new insights into schizophrenia treatment. In line with Guang Zhi et al., who discovered that rutin reduces neuroinflammation and provides neurological protection in a laboratory rat model for subarachnoid hemorrhage, presumably by reducing the inflammatory signaling pathway^75^. As a result, our data indicated that rutin may provide therapeutic benefits by decreasing the inflammatory response (Fig. 5). IL-6 dysregulation can cause abnormalities in neurotransmitter systems, including dopamine and glutamate that are important in the mental signs of schizophrenia. Studies are looking at how IL-6 controls these disturbances, which contribute to prefrontal brain dysfunction and decline in cognition^76^. Although the CPZ model is generally used to investigate demyelination and remyelination, its application to schizophrenia stems from its capacity to imitate white matter reduction, dopamine, glutamate and GABA malfunction^49^. Our findings revealed that CPZ significantly increases dopamine and glutamate while decreasing NMDA brain content. The cognitive and negative symptoms of schizophrenia are thought to be caused by glutamate dysregulation, specifically NMDA receptor hypofunction. Increased glutamate levels can potentially cause excitotoxicity and neuronal injury. Disruptions in dopamine-glutamate communication may drive schizophrenia pathophysiology, resulting in abnormally high striatal dopamine levels. Furthermore, increased midbrain dopamine neuron activation resulted in striatal dopamine elevations and was linked with positive signs of schizophrenia (hallucinations and delusions)^77^. CPZ significantly (*p* < 0.05) increased dopamine by 263% (0.29 ± 0.02 vs. 0.08 ± 0.002) and glutamate by 6.5% (1.79 ± 0.01 vs. 1.68 ± 0.01) while decreasing NMDA by 20.1% (1.74 ± 0.02 vs. 2.18 ± 0.06). Our findings exhibited that free rutin, F4, and coated CF4 rutin treatments may have therapeutic effects on the dopamine and glutamate pathways by lowering dopamine by 12.3%, 23.4% and 55.5% (0.25 ± 0.01, 0.22 ± 0.01, 0.13 ± 0.03 vs. 0.29 ± 0.02) and glutamate excitotoxicity by 4.7%, 5.2%, and 6.5% (1.71 ± 0.0012 1.70 ± 0.0001 1.68 ± 0.0007 vs. 1.79 ± 0.01) and increasing NMDA brain content by 6%, 11% and 23% (1.84 ± 0.01, 1.93 ± 0.01, 2.14 ± 0.01 vs. 1.74 ± 0.02) respectively, as compared to the CPZ group. Consistent with earlier studies, Rutin was found to prevent seizures in kainic acid-treated rats. It achieves this effect by lowering levels of glutamate, reducing inflammation, and protecting against neuron death^78^.

NRG1 is a crucial factor in brain growth and normal function, acting as a developmental regulator that also shields the fetal brain's white matter from injury. It is necessary for the development and differentiation of neurons, astrocytes, and oligodendrocytes^79^. NRG1 expression changes are linked to anomalies in neurotransmission, particularly in the glutamate and dopamine systems, both of which are implicated in schizophrenia^80^. In the present work, we discovered that NRG1 was downregulated in CPZ-treated mice. This finding is consistent with a previous study, which discovered that CPZ caused a significant reduction (*p* < 0.05)in hippocampus NRG-1 protein by 559% (98.49 ± 6.51 vs. 649.25 ± 63.14)^81^. In the current investigation, free rutin, F4, and coated CF4 rutin treatments caused an elevation in NRG1 brain content reaching 210%, 366%, and 497% (305.28 ± 14.98, 459.26 ± 13.58, 587.86 ± 32.58 vs. 98.49 ± 6.5), respectively, as compared to CPZ group. Our study is the first to show the action of rutin on NRG1 and related pathways, contributing to neuroplasticity and myelination, as well as its neuroprotective role in countering oxidative stress and neuroinflammation, which are significant in schizophrenia pathophysiology. NRG1 signaling may influence myelination, as it is involved in oligodendrocyte function. As a result, changes in NRG1 may cause aberrant myelination, as evidenced by MBP brain content. The CPZ model suggests that these changes in energy metabolism contribute to oligodendrocytes apoptosis and subsequent demyelination. CPZ administration in animals leads to inhibition of myelin-related genes and proteins, such as (MBP, consequent demyelination (myelin loss) and myelin breakdown in the brain, and loss of oligodendrocytes that are responsible for creating myelin. Because of this damage, this animal model displays behavioral symptoms similar to those of human schizophrenia patients, including deficits in working memory^79^. These results confirmed that coated F4 nano-formula protects against prenatal brain white matter injury via regulation of NRG1/MBP, which in turn maintains the glutamate and dopamine neurotransmitters.

The blend coating is assumed to improve activity. Glucosamine has been reported to act as a potential delivery ligand and transport mediator for drug delivery across the BBB^82^. Glucosamine-coated non-ionic surfactant vesicles are transported via glucose transporter 1 (GLUT1)-mediated transcytosis, delivering payloads into brain cells and enhancing treatment outcomes in viral encephalitis models^34,35^. Researchers developed glucosamine-functionalized micelles and dendrimers that improved brain permeation and delivered drugs to glioma cells more efficiently than non-functionalized carriers. Another study found that poly(d-glucosamine)-coated lipidic nanovesicles improved cerebral uptake and prolonged the release of anticancer agents for glioblastoma therapy^36^. Also, metabolic imaging using advanced MRI showed that glucosamine crossed the BBB via uptake and metabolism in various brain regions, supporting its use as both a metabolic biomarker and a transport vector^83^. Thus, as inferred from previous studies, glucosamine functionalization may improve drug delivery to brain tissues. This makes it a useful tool to develop therapies for central nervous system disorders.

### Histopathological findings

**Fig. 6:**
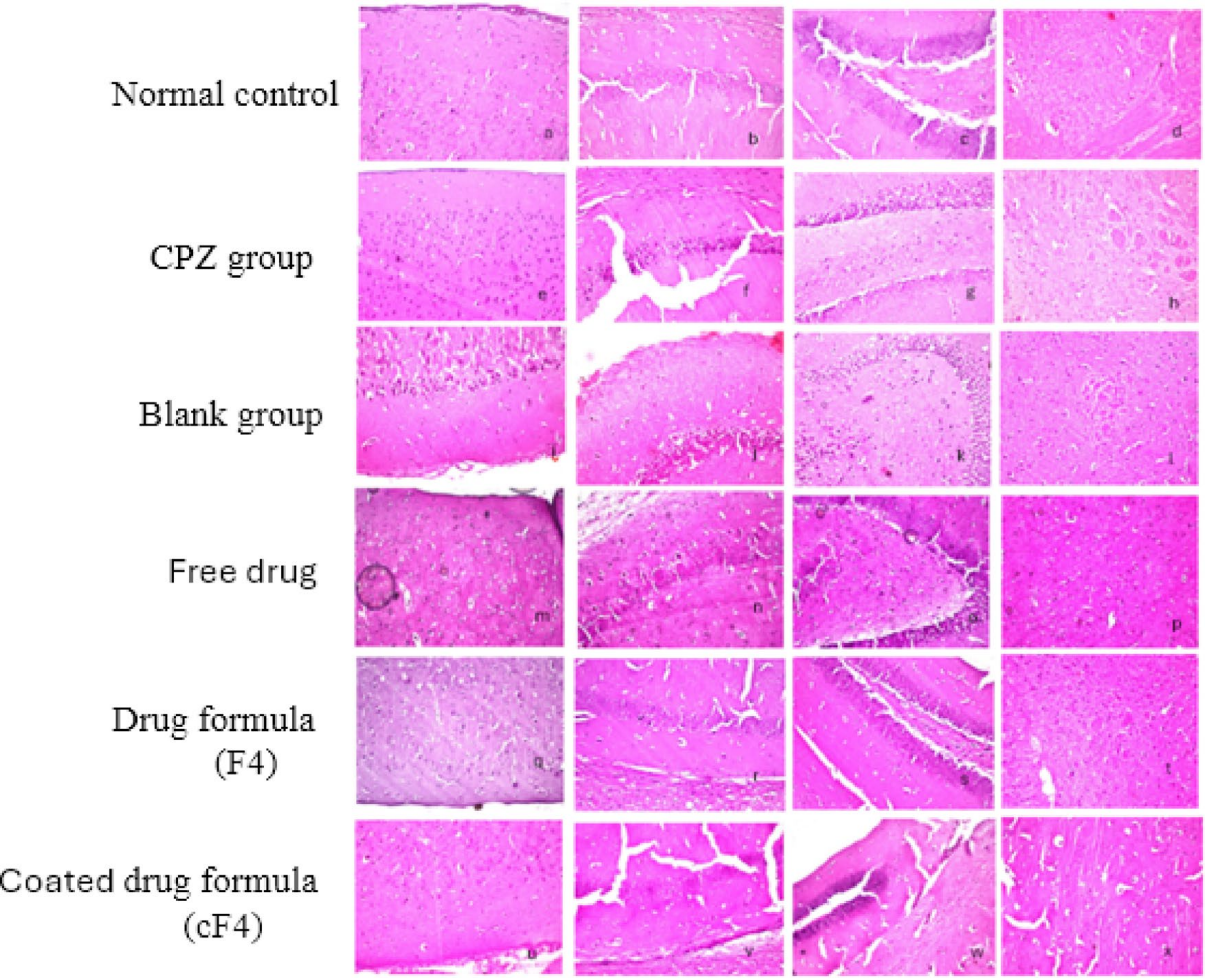
Effect of the free rutin, uncoated F4, and coated CF4 nanoformulations on histopathological findings of the brain on CPZ model in mice. Normal control cerebral cortex (**a**), hippocampus (subiculum & fascia dentata and hilus) (**b**,**c**), and striatum (**d**). CPZ group cerebral cortex (**e**), hippocampus (subiculum & fascia dentata and hilus) (**f**,**g**), and striatum (**h**). Blank group cerebral cortex (**i**), hippocampus (subiculum and fascia dentata and hilus) (**j**,**k**), and striatum (**l**). Free cerebral cortex (**m**), subiculum (**n**), fascia dentata and hilus (**o**), and striatum (**p**). Drug formula (F4) cerebral cortex (**q**), subiculum & fascia dentata and hilus (**r**,**s**) and striatum (**t**). Drug-coated formula (CF4) cerebral cortex, subiculum, fascia dentata, and hilus (**u**–**w**) and striatum (**x**). H &E × 16.

No histopathological alterations were observed in the cerebral cortex of the normal control group (Fig. 6a), hippocampus (subiculum & fascia dentata and hilus) (Fig. 6b,c), as well as striatum (Fig. 6d). However, the CPZ-treated group exhibited nuclear pyknosis and degenerative changes in most neurons of the cerebral cortex (Fig. 6e), hippocampus (subiculum and fascia dentata and hilus (Fig. 6f,g), In addition, the striatum showed multiple eosinophilic plaque formations accompanied by nuclear pyknosis and neuronal degeneration (Fig. 6h). Also, blank group showed nuclear pyknosis and degeneration in all the neurons of the cerebral cortex (Fig. 6i), hippocampus (subiculum and fascia dentata and hilus) (Fig. 6j,k). In addition, the striatum exhibited nuclear pyknosis and degenerative changes in most neurons, along with focal plaque formation (Fig. 6l). Free drug showed no histopathological alteration in cerebral cortex (Fig. 6m), normal histological structure of the neurons in subiculum (Fig. 6n), nuclear pyknosis and degeneration of the neurons in fascia dentata and hilus (Fig. 6o), and nuclear pyknosis and degeneration in some neurons in striatum (Fig. 6p). The drug formula F4 exhibited nuclear pyknosis and degeneration of a few neurons in the cerebral cortex (Fig. 6q), no histopathological alteration in the subiculum & fascia dentata and hilus (Fig. 6r,s). The striatum exhibited focal eosinophilic plaque formation accompanied by nuclear pyknosis and neuronal degeneration (Fig. 6t). The rutin-coated nanoformula CF4 group showed no histopathological alterations in the cerebral cortex, subiculum, fascia dentata, or hilus (Fig. 6u–w). Moreover, the striatum exhibited no histopathological alteration (Fig. 6x). According to previous studies, rutin has been shown to alleviate several types of secondary brain injuries, including brain edema, BBB disruption, neurological deficits, and neuronal death^84^. The histopathological examination revealed improved neuroprotective and remyelination effects in the CF4-treated group compared to F4- and free rutin-treated groups. The beneficial properties of both glucosamine and chitosan contributed to this improvement.

**Fig. 7:**
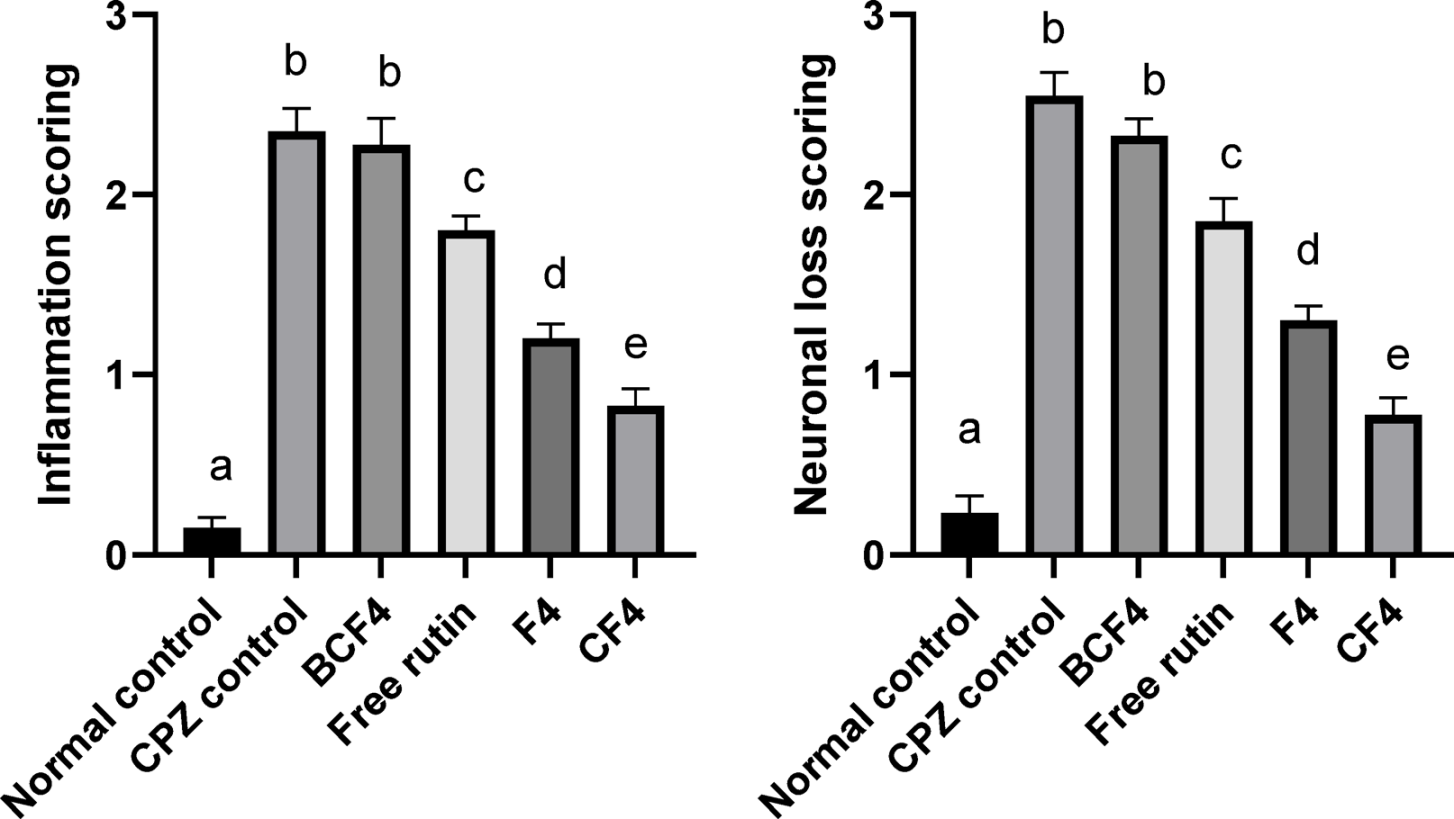
Effect of the free rutin, uncoated F4, and coated CF4 nanoformulations on histopathological scoring. Identical letters indicate no significant difference between parameters, whereas differing letters signify a significant difference at *p* < 0.05.

The severity of brain alterations was evaluated microscopically in a blinded manner. The histopathological scoring of the stained brain tissues is presented in Fig. 7. Collectively, both histological and biochemical results implied that CF4 was more effective than F4 and free rutin. The biochemical analysis implied better improvement for CF4, evidenced by reduced levels of glutamate, dopamine, and IL-6, and elevated levels of MDA, NRGα, and MBP. This enhancement can be attributed to its nanosize, accompanied by its modified surface properties credited to the role of the functional coating, which can improve absorption due to the well-known bioadhesive properties of chitosan and the reported capabilities of glucosamine to circumvent BBB, thus highlighting the potential to improve schizophrenia-like behavioral alterations, pathophysiologic, and histopathologic manifestations in the used animal model.

Careful selection of formulation parameters may play an integrated, key role in augmenting absorption, cellular internalization, and brain-delivery potential. The nanometric architecture of the hybrid system that comprised the biocompatible PCLand Geleol™. Also, the surfactant, Tween® 80, was previously reported to have brain-targeting capabilities and assist penetration through the BBB^54^. In addition, research has indicated that Tween 80 can impede exocytosis and drug efflux of drugs by inhibiting p-glycoprotein^85^. Also, the surface modification with the chitosan-glucosamine blend may enhance bioadhesive properties and receptor affinity. This can promote nanoparticle interactions with brain endothelial cells and assist in crossing the BBB. Thus, lowering behavioral, neurochemical, and histopathological abnormalities associated with the disorder.

While current results support the functionalized rutin-loaded formulation's enhanced effect, its ability to target and be localized within brain tissue remains to be further confirmed. The main limitation is the absence of comprehensive pharmacokinetic and biodistribution data, which are essential for fully interpreting the in vivo behavior of the developed formulation. Future detailed pharmacokinetic and biodistribution assessments are required for the precise estimation of brain targeting.

## Conclusion

Rutin-loaded hybrid nanoparticles were efficiently formulated, combining polycaprolactone and Geleol™ within a single system, utilizing the emulsion solvent evaporation technique. The selected optimized formulation (F4) was surface engineered by applying a blend of chitosan hydrochloride and glucosamine as a functional coating to enhance bioadhesion, cellular interaction, and potential brain delivery (CF4). The therapeutic efficacy of free rutin, F4, and CF4 was assessed using CPZ-induced schizophrenia in mice by biochemical and histopathological examinations. The results demonstrated therapeutic improvements, such as reduced neuroinflammation, increased brain content of MBP and NRG1, and restoration of myelination. In addition, it showed modulation of dopamine and glutamate pathways, potentially alleviating some of the cognitive and neurochemical imbalances associated with schizophrenia. The behavioral, biochemical, and histopathological investigations depicted that the proposed system improved the therapeutic effect of rutin. Moreover, the innovative surface-functionalized blend of chitosan and glucosamine in the CF4 formula improved the effectiveness compared with F4 and free rutin. This work provided promising therapeutic outcomes for the surface functionalized rutin-loaded hybrid nanoparticles. The results bring up new avenues for non-invasive neurological treatments. However, the study was confined to an experimental CPZ-induced schizophrenia mouse model, which has its limitations. It must be acknowledged that no single animal model can replicate all disease manifestations. Thus, the findings need to be translated to clinical settings. Further investigations can be conducted to better evaluate the effectiveness, safety, and translational applicability of the proposed formulation for human use. Future studies will involve detailed pharmacokinetic profiling, analysis of brain biodistribution, in vivo real-time imaging, and long-term toxicity assessments. This can facilitate the development of safe and effective schizophrenia therapy for patients.

## Data Availability

All data generated or analyzed during this study are included in this published article.
